# A Retrospective Analysis of Temporal Lobe Gliosis after Middle Fossa Resection of Small Vestibular Schwannomas

**DOI:** 10.3390/brainsci14030295

**Published:** 2024-03-20

**Authors:** Matthias Scheich, Miriam Bürklein, Manuel Stöth, Brigitte Bison, Rudolf Hagen, Stephan Hackenberg, Marius L. Vogt

**Affiliations:** 1 Department of Oto-Rhino-Laryngology, Plastic, Aesthetic and Reconstructive Head and Neck Surgery, University Hospital of Würzburg, Josef-Schneider-Str. 11, 97080 Würzburg, Germany; buerklein_m@ukw.de (M.B.); stoeth_m@ukw.de (M.S.); hackenberg_s@ukw.de (S.H.); 2 Department of Diagnostic and Interventional Neuroradiology, Faculty of Medicine, University of Augsburg, Stenglinstr. 2, 86156 Augsburg, Germany; brigitte.bison@uk-augsburg.de; 3 Department of General Practice, University Hospital of Würzburg, Josef-Schneider-Str. 2, 97080 Würzburg, Germany; hagen_r@ukw.de; 4 Department of Diagnostic and Interventional Neuroradiology, University Hospital of Würzburg, Josef-Schneider-Str. 11, 97080 Würzburg, Germany; vogt_m2@ukw.de

**Keywords:** vestibular schwannoma, acoustic neuroma, middle fossa approach, gliosis

## Abstract

Introduction: The middle cranial fossa (MCF) approach is a well-established procedure in surgery of the internal auditory canal, as well as with the retrosigmoid and translabyrinthine approaches. It is commonly used in the hearing-preserving microsurgery of small vestibular schwannomas (VS). The debate about the “best” approach for the microsurgery of small VS without contact to the brainstem is controversial. It has been stated that the MCF approach leads to irreversible damage to the temporal lobe, which may be evident in follow-up magnet resonance imaging (MRI) as gliosis in up to 70% of patients. Materials and Methods: This study represents a retrospective chart analysis conducted at a tertiary university hospital. Here, 76 postoperative MRIs were re-evaluated by an experienced neuroradiologist and compared with the preoperative images. Temporal lobe gliosis was classified on an ordinal scale as absent, slight, moderate or severe. Occurrence of gliosis was matched to the clinical predictors (tumor stage, tumor volume, sex, age, and side). Results: No case of severe or moderate gliosis was found in the patient group. Slight gliosis of the temporal lobe was rare and was only detected in four patients (5%). There was no relation between clinical predictors and the incidence of gliosis. Conclusions: In our cohort, postoperative MR imaging did not reveal relevant damage to the temporal lobe parenchyma. This confirms the safe concept of microsurgery of small tumors via the middle fossa approach. The severe glioses described in other studies may be caused by a forced insertion of the retractor or by more extended approaches. However, further prospective neurocognitive studies seem to be necessary in order to assess functional changes in the temporal lobe.

## 1. Introduction

The middle cranial fossa (MCF) approach is a well-established procedure in surgery of the internal auditory canal (IAC). It is an extradural approach that was first used more than 130 years ago [1], but its widespread use began with the description by William House in 1961 [2]. It has undergone several modifications (e.g., by Fisch [3] or Brackmann [4]). In addition to the neurosurgical retrosigmoid (RS) access and destructive translabyrinthine (TL) approaches, the MCF approach is mainly used by otosurgeons for hearing-preserving microsurgery of small vestibular schwannomas (VS). The functional outcomes are good [5,6,7] and complication rates are low [8]. Other indications include vestibular neurectomy, petrous apex biopsy, cholesterol granuloma, or decompression of the internal auditory canal in patients with neurofibromatosis type II.

The discussion about the optimal approach for microsurgery of small T1/T2 VS without contact to the brainstem (Koos [9] grading system: T1 = intracanalicular, T2 = with extrameatal extent but without contact to the brainstem) is still controversial. The functional outcomes and complications of both the MCF and RS approaches are comparable and mostly dependent on the expertise of the center or surgeon [10]. Middle fossa surgery involves elevating the temporal lobe with a retractor to access the petrous bone and the IAC from superior. However, it has repeatedly been claimed that the required elevation of the brain may result in irreversible damage to the temporal lobe. According to Schick et al. this damage would present as gliosis in follow-up magnetic resonance imaging (MRI) in up to 70% of patients [11]. Although there was very little literature on that subject published in the early 2000s [11,12,13], this claim has continued to persist in discussions at international conventions, such as most recently at the 9th Quadrennial International Conference on Vestibular Schwannoma held in Bergen, Norway, in 2023.

Gliosis formation can be triggered by any injury of the brain like inflammation, stroke, or trauma. This highly complex process is also called reactive gliosis and is caused by the cytokine-mediated activation and proliferation of glial cells, predominantly driven by astrocytes, with upregulation of glial fibrillary acidic protein (GFAP). Reactive Gliosis results in an astrocyte scar and consists of three different compartments: a central core of non-neural tissue containing fibroblasts, fibrocytes, and an extracellular matrix; a compact astrocyte scar surrounding the core with densely packed astrocytes and perilesional viable neural tissue consisting of neurons, oligodendrocytes, and astrocytes [14,15].

Postoperative gliosis is seen as a focal signal increase adjacent to the petrous bone in T2-weighted and as reduced signal in T1-weighted MRI sequences, whereas T2 weighted sequences, with “inversion recovery” being the most sensitive for the detection of gliotic changes. The formation of gliosis is completed in most cases by two to four weeks after the initial incident [14].

This study aims to show that MRI changes in postoperative imaging are rare and much less pronounced than suspected. We interpret historical data and place it in the context of our own findings. Furthermore, we try to identify clinical predictors for the occurrence of postoperative gliosis.

## 2. Materials and Methods

### 2.1. Patient Data

In this retrospective study, we reviewed the cases of 93 consecutive patients who underwent microsurgery for unilateral vestibular schwannoma (VS) in our department between January 2012 and November 2015. In total, 76 patients with sufficient imaging were included in the analysis, whereas 17 had to be excluded due to missing postoperative imaging. There were 33 male and 43 female patients, aged 16 to 75 years (mean 51). Tumor stages were T1 (intracanalicular) in 36 cases and T2 (extrameatal extent without contact to the brainstem) in 40 patients. Morever, 46 tumors were located on the right side and 30 on the left side. The mean tumor volume was 176 mm^3^ (range 19–801 mm^3^).

### 2.2. Surgery

All of the patients underwent total resection of the VS via the MCF approach by the same skullbase surgeon, as previously described in detail [16,17]. Patients were placed in a supine position, a Mayfield head holder was not required. After making a curved skin incision, a flap was prepared from the temporalis muscle. A craniotomy of approximately 4 cm × 3 cm was then drilled out of the temporal bone 1 cm superior to the zygoma, positioned with 2/3 anterior and 1/3 posterior to the outer ear canal. While drilling the craniotomy, 250 mL of mannitol (20%) was administered to reduce intracranial pressure. The dura of the middle fossa was separated from the petrous bone and a self-retaining Fisch retractor was gently placed to elevate the temporal lobe. The tip of the blade was placed as far as the posterior edge of the petrous bone, where the porus of the IAC was suspected. It was important to ensure that the retractor was not placed too far posteriorly in order to avoid pressure on the inferior anastomotic vein (vein of Labbé), and thus avoid possible complications such as impairment of the venous drainage or congestive hemorrhage. After that, the canal of the greater petrosal nerve, the arcuate eminence, and the superior petrosal sinus were identified. The blue line of the superior semicircular canal (SSC) was exposed. After drilling off the roof of the IAC, the dura was opened to release CSF and further reduce intracranial pressure. The dura was then incised and dissected up to the cerebellopontine angle (CPA). The tumor was exposed for debulking and complete resection. In most cases, debulking was performed with a hand-held non-contact CO_2_ laser (Omniguide Inc., Cambridge, MA, USA). After tumor removal, 250 mg of prednisolone was administered intravenously and this was repeated on the first postoperative days. Then, the IAC was closed using a temporalis muscle graft and fibrin glue. Opened temporal bone cells were meticulously sealed with wax or a fibrin sealant patch. Finally, the bone flap was replaced and the wound was sutured.

The surgical procedure time was defined as the time from the incision to the end of wound closure. The mean time was 164 min (median: 159 min; range: 91–302 min).

### 2.3. Imaging

In all patients, preoperative imaging was discussed in our interdisciplinary skull base conference prior to decision making. The MRIs of all 76 patients with adequate follow-up imaging were re-evaluated by an experienced neuroradiologist and compared to the preoperative imaging. Here, 61 of 76 (80.3%) MRI postoperative examinations included a T2 weighted sequence with “inversion recovery” like T2 FLAIR (fluid attenuated inversion recovery) or TIRM (turbo-inversion recovery-magnitude). The mean interval between surgery and the first follow-up MRI was 11.9 months (median: 11.7 months; range 3.2–30.5 months). Temporal lobe gliosis was classified as absent, slight, moderate, or severe according to the criteria in Table 1.

### 2.4. Predictors

To determine whether the occurrence of gliosis was influenced by predictors, we correlated it with T-stage, tumor volume, surgical procedure time, sex, tumor laterality (right/left), and age in all 76 cases. Age, surgical procedure time, and tumor volume were dichotomized near the median.

### 2.5. Statistics

All statistical analyses were performed with the Statistical Package for Social Sciences (SPSS, IBM SPSS Statistics 29, Chicago, IL, USA). Predictors were analyzed using Pearson’s chi-squared test. A significance level of *p* < 0.05 was used to determine significance.

## 3. Results

Postoperative MRI scans did not reveal any degree of gliosis in the temporal lobe in 72 patients. There was no severe or moderate gliosis in all 76 patients. Slight gliosis of the temporal lobe was rare and was only detected in four patients (Table 2).

**Figure 1:**
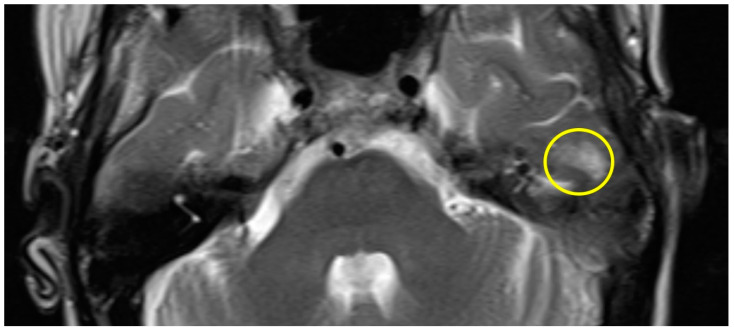
Axial T2w MR imaging of patient #1 (yellow circle = gliosis). Supplementary MR imaging can be downloaded.

**Figure 2:**
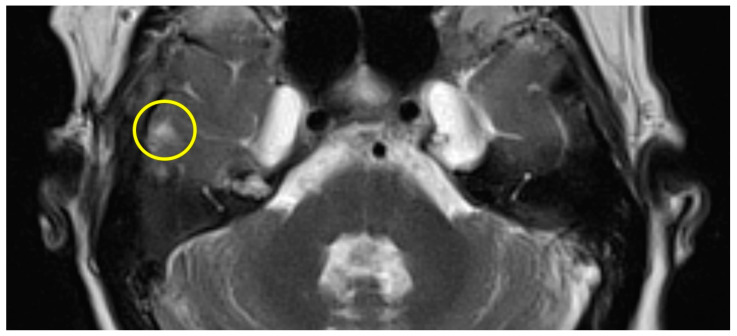
Axial T2w MR imaging of patient #2 (yellow circle = gliosis). Supplementary MR imaging can be downloaded.

Patient #1 exhibited a slight gliosis on the left side during the follow-up MR 12 months postoperatively. On axial T2 weighted slides, a focal cortical and subcortical T2w signal increase was observed in the left temporal lobe (Figure 1). Patient #2 received early MR imaging only 3 months after surgery, which did not reveal any abnormalities. The subsequent second postoperative MR was conducted 16 months after surgery, revealing a slight gliosis of the right temporal lobe on the T2 axial images (Figure 2).

**Figure 3:**
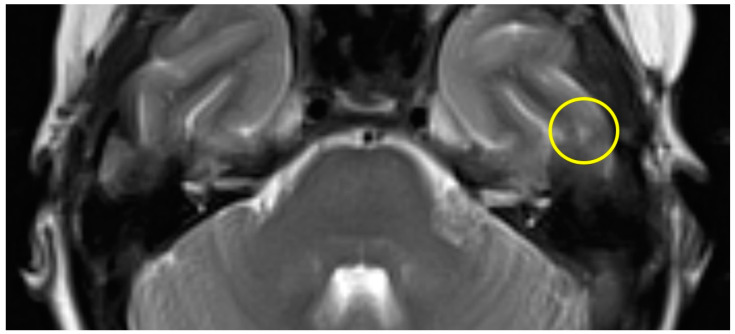
Axial T2w MR imaging of patient #3 (yellow circle = gliosis). Supplementary MR imaging can be downloaded.

**Figure 4:**
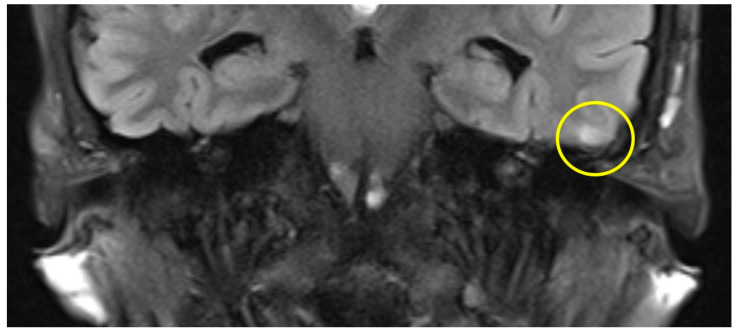
Coronal T2 flair MRI of patient #4 (yellow circle = gliosis). Supplementary MR imaging can be downloaded.

The first postoperative MR imaging of patient #3 was made 11 months after surgery and showed a very discrete focal hyperintensity on axial T2w imaging in the left temporal lobe (Figure 3). In patient #4 (Figure 4), a slight rim of gliosis was detectable on the left side in the follow-up imaging 12 months postoperatively, which can be seen in coronal T2w FLAIR (Fluid-Attenuated Inversion Recovery) imaging.

For further statistical analysis, the cohort was dichotomized into two groups depending on age: patients older than 52 years (*n* = 39; 3 glioses) and patients with 52 years of age or younger (n = 37; 1 gliosis). Although gliosis was less frequent in younger patients, this difference did not reach statistical significance (*p* = 0.355). There was no significant correlation between tumor dimensions and gliosis in terms of T-stage: 3 glioses were found in 40 T2 tumors and one case in 36 T1 tumors (*p* = 0.357). Tumor volume (>140 mm^3^) was not predictive as well (*p* = 0.304). All four gliosis occurred in women, but we did not find significant differences in correlation to sex (*p* = 0.070) nor to tumor laterality (3/30 left ears vs. 1/46 right ears; *p* = 0.135). Regarding surgical procedure times, three gliosis occurred in patients operated on for less time than the mean time of 164 min. There was no significant correlation (*p* = 0.330) with the duration from incision to closure (dichotomized at the median; <159 min (n = 39) versus ≥159 min (n = 37)).

## 4. Discussion

The middle cranial fossa approach is a well-established and safe option for hearing preservation microsurgery in small vestibular schwannomas. In 2003, Brors et al. [12] first reported on postoperative MRI findings after middle fossa and translabyrinthine VS resection. Their primary goal was to check for residual/recurrent tumor on postoperative MR imaging. Additionally, they conducted an evaluation of temporal lobe gliosis and changes in fat grafts. In 48 MCF cases operated on between 1992 and 1998, they found 15 glioses of different degrees (31%). They did not provide specific information on the tumor size, degrees of gliosis, or intraoperative application of mannitol. Two years later, Minovi et al. [13], from the same department, re-evaluated 89 MCF cases from a period spanning 1988–2004. In the larger series, they found even more cases of temporal lobe gliosis (41%). Once again, the authors did not refer to the degrees of gliosis or the intraoperative use of mannitol. In 2008, Schick (who was also the senior author of the Brors paper) et al. [11] evaluated 32 patients of another department one year after MCF resection of VS. They found gliosis in 22 MRIs (69%), classified as slight in 11 cases, moderate in 9, and severe in 2 cases.

In neuroradiological literature, the occurrence of gliosis after middle fossa resection is mentioned, but it is often cited secondarily [18,19] from Schick et al.’s work [11]. Other publications discuss postoperative imaging after VS surgery [20,21,22], but primarily focus on detecting recurrence and changes in the IAC, without mentioning temporal lobe changes or gliosis in general. Severe damage to the temporal lobe, which were already detected in cranial computed tomography on the first postoperative day, were excluded by Stripf et al. [23] in an analysis of 62 MCF surgeries for VS.

In our retrospective analysis, we did not observe any relevant damage to the temporal lobe. We found four cases of slight gliosis (5%) in MR imaging and no patients with moderate or severe gliosis, respectively. Hence, gliosis was less frequent and less severe in our study than it was proclaimed in the three studies from the 2000s [11,12,13]. This difference may be due to the less radical and gentler placement of the dura retractor, which is nowadays more common than in the 1990s. Furthermore, we consequently administered mannitol before craniotomy to reduce intracranial pressure, as was also done by Schick, but not mentioned by Brors or Minovi. Another point to consider is the selection of tumors and the resulting extent of surgery. Minovi et al. also operated on Wigand Class C tumors (equivalent to Koos T3) in 12% of cases, whereas we only included T1 and T2 cases. Schick et al. used an enlarged MCF (EMCF) approach that also allows for the resection of larger tumors with an extrameatal extent of more than 2 cm [24]. It is not further explained if this extension of the access was necessary in all cases and how the distribution of tumor sizes was in their patients. Larger tumors are typically associated with more manipulation and a higher rate of complications (such as TIA, meningitis, and epilepsy) [8,11,25].

In addition, we retrospectively analyzed predictors of the four cases with (slight) gliosis in our series. The wider extension at the porus of the IAC, which is necessary for tumors with extrameatal extent, may also result in higher pressure on the retractor. However, we did neither find significant differences between T1 or T2 tumors nor between higher or lower tumor volumes. Although the dura is more fragile and less elastic in elderly patients [26,27], we did not find any evidence to suggest that elderly patients are more susceptible to experience damage. The duration of the retraction applied may impact on the occurrence of glioses. None of the three groups with more frequent glioses reported surgical procedure times. Unfortunately, we did not document the exact time of the retractor in place. However, we analysed the total surgical time from incision to skin closure, which might at least give approximate information about the exposure time. Detailed information on surgical procedure times in VS surgery and particularly in MCF approaches, is limited and inconsistent in the literature (230 [28], 339 [29] and 418 [30] minutes respectively). Reports often fail to clearly define, whether they refer to skin-to-skin time or to time, including the preparation for monitoring. Our skin-to-skin time of 159 min appears to be shorter than in comparable cases, but is durable, as we reported in 2012 in a series of 40 selected patients [31]. Furthermore, we were unable to identify any association with sex or tumor laterality. All this may be due to our relatively homogeneous cohort of small tumors and the exclusion of T3 tumors.

## 5. Limitations

This study is of course just a retrospective study with no comparison groups (e.g., translabyrinthine or retrosigmoid approach). Additionally, the study only examines changes in postoperative MR imaging, which are not always related to complaints or possible neurocognitive deficits. Furthermore, there was no standardized MR protocol for post-surgery MR imaging, so gliotic changes might be underdetected. Only 80% of the postoperative MRI examinations contained a T2 weighted sequence with “inversion recovery”, which is most sensitive for the detection of gliotic areas. The sensitivity for very discrete gliotic changes might be higher if high-resolution isotropic 3D MR imaging had been performed.

## 6. Conclusions

In summary, our series of 76 patients did not show relevant damage to the temporal lobe after middle fossa resection of small T1/T2 Vestibular Schwannomas. We found that temporal lobe gliosis was not as frequent as previously thought. Therefore, the MCF approach should not be discredited based on a small number of historic data from overlapping authors and overlapping patient groups.

Of course, further prospective neurocognitive studies are necessary to reliably assess functional changes in the temporal lobe or to compare MCF with TL and RS approaches.

## Figures and Tables

**Table 1:** Radiological classification of the extent of temporal lobe gliosis (adopted from [11]).

none (0)	
slight (1)	parts of the inferior temporal gyrus, less than half of the thickness (cranial-caudal)
moderate (2)	further parts of the inferior temporal gyrus
severe (3)	more than one gyrus

**Table 2:** Characteristics of 4 patients with gliosis (f = female; min = minutes).

	Tumor Stage	Sex	Age	Laterality	Time Interval between Surgery and MRI	Incision-to-Closure Time	Extent of Gliosis
Patient #1	T2	f	58	left	12 months	175 min	slight
Patient #2	T2	f	62	right	16 months	159 min	slight
Patient #3	T2	f	57	left	11 months	131 min	slight
Patient #4	T1	f	49	left	12 months	159 min	slight

## Data Availability

All data of the study is available upon request in the Department of Oto-Rhino-Laryngology, Plastic, Aesthetic and Reconstructive Head and Neck Surgery at the University Hospital of Würzburg, Germany. The data are not publicly available due to specific ethical and privacy considerations.

## Supplementary Materials

The following supporting information can be downloaded at: https://www.mdpi.com/article/10.3390/brainsci14030295/s1. “Patient 1 supplemetary MR imaging”; “Patient 2 supplemetary MR imaging”; “Patient 3 supplemetary MR imaging”; “Patient 4 supplemetary MR imaging”.
