# Supplementary figures and images for: A Retrospective Analysis of Temporal Lobe Gliosis after Middle Fossa Resection of Small Vestibular Schwannomas
Source: Brain Sci. 2024 Mar 20;14(3):295. doi: 10.3390/brainsci14030295 (PMC10969293; doi:10.3390/brainsci14030295)

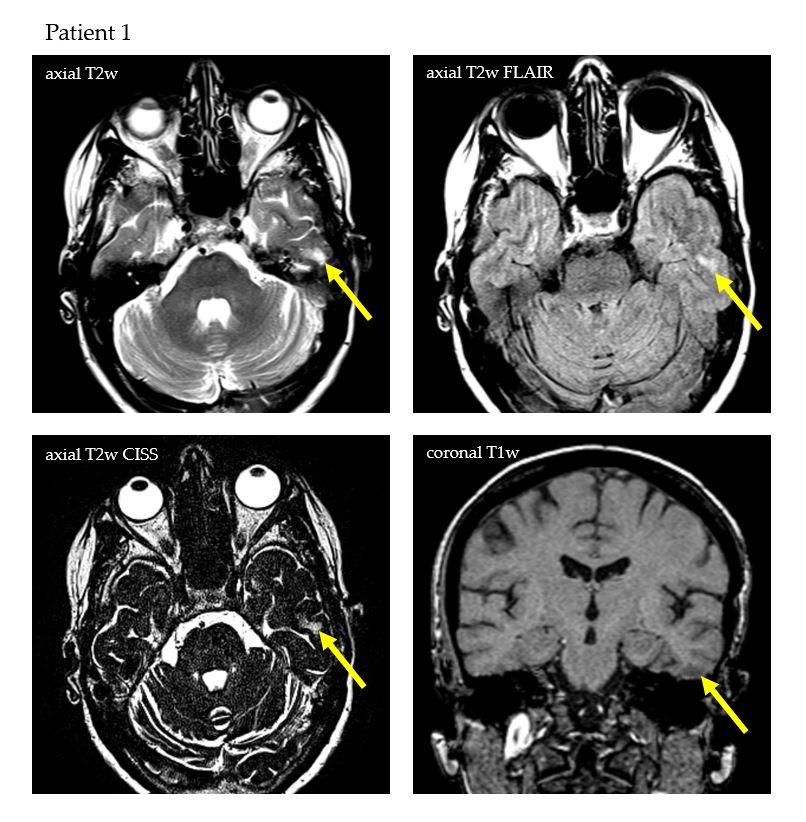

Supplement: Supplementary file 1 [file brainsci-14-00295-s001.zip › Patient 1 supp.jpg]

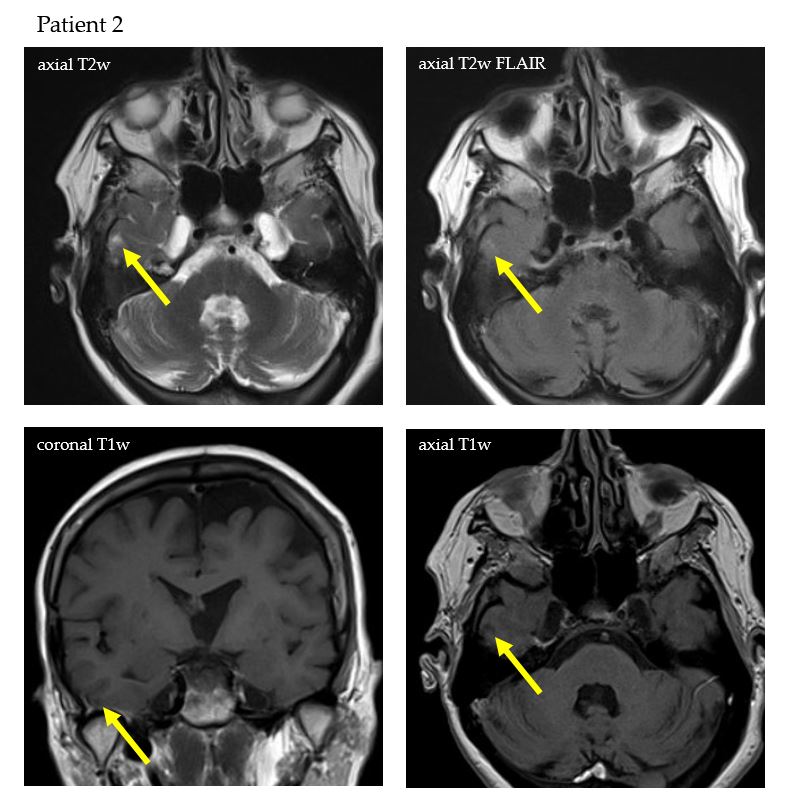

Supplement: Supplementary file 1 [file brainsci-14-00295-s001.zip › Patient 2 supp.JPG]

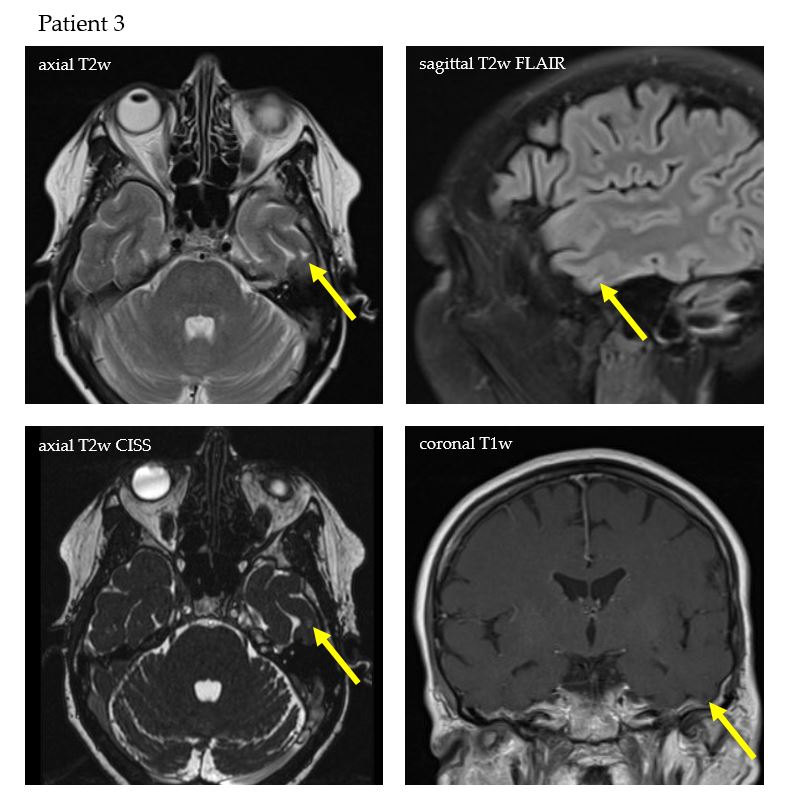

Supplement: Supplementary file 1 [file brainsci-14-00295-s001.zip › Patient 3 supp.JPG]

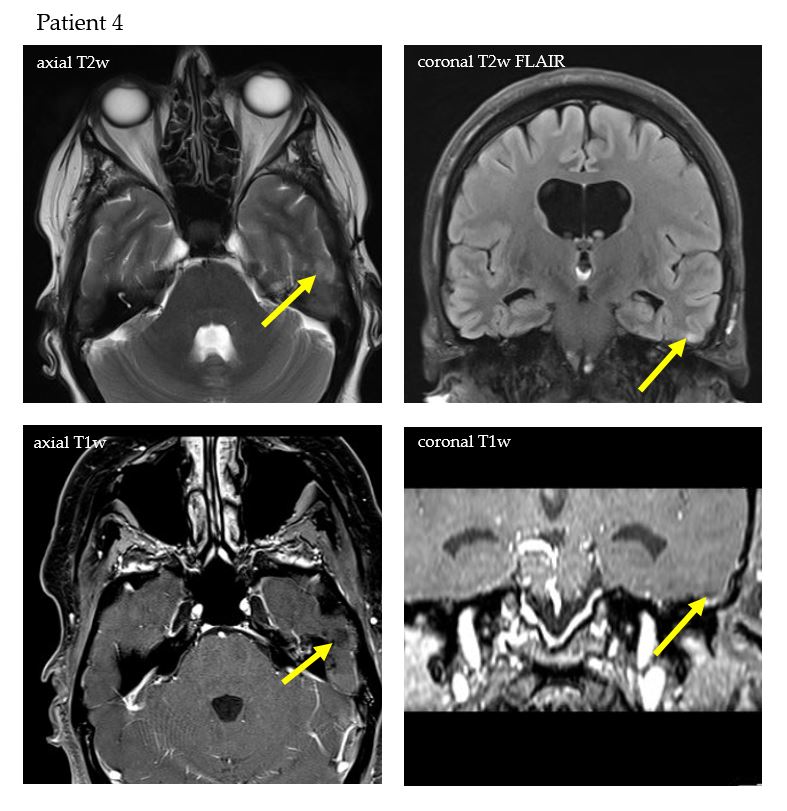

Supplement: Supplementary file 1 [file brainsci-14-00295-s001.zip › Patient 4 supp.JPG]
